# Severe asterixis due to hypermagnesemia in chronic renal failure: a case report

**DOI:** 10.1007/s10072-020-04945-x

**Published:** 2021-01-06

**Authors:** Mitsunori Morimatsu, Kaoru Ono, Akira Harada

**Affiliations:** 1 Division of Neurology, Tokuyama Medical Association Hospital, Shunan, Japan; 2 Ono Clinic, Shunan, Japan

## Introduction

Asterixis is a type of negative myoclonus characterized by sudden irregular lapses of posture of various body parts due to involuntary interruption in muscle contraction [1, 2]. Flapping tremor or liver flap, a type of asterixis, associated with liver disease is well-known. However, it occurs in many disorders systemically in impaired visceral functions, drug intoxications, and dyselectrolytemia, and unilaterally in focal brain lesions (Table 1) [1]. Therefore, full examinations are necessary for detecting the causative disorders.

**Table 1:** Causes of asterixis [1]

Bilateral asterixis	Unilateral asterixis
Metabolic: liver failure, azotemia, respiratory failure	Focal brain lesions at:
Drugs:	Thalamus
Sedatives: benzodiazepines, barbiturates	Corona radiata
Anticonvulsants: phenytoin (phenytoin flap), carbamazepine, valproic acid, gabapentin	Anterior cerebral artery territory
Antipsychotics: lithium	Primary motor cortex
Antibiotics: ceftazidime	Parietal lobe
Others: metoclopramide	Cerebellum
Dyselectrolytemia: hypomagnesemia, hypokalemia	Midbrain
Bilateral structural brain lesions	Pons

As dyselectrolytemia causing asterixis, hypomagnesemia, and hypokalemia have been described [1, 2]. With reference to hypermagnesemia, it causes depression of nerve and muscle functions consisting of decreased tendon reflexes, muscle flaccid paralysis, and impaired consciousness terminating in coma [3]. To the best of our knowledge, there have been no case reports showing asterixis due to hypermagnesemia. This is a rare case who exhibited severe asterixis due to hypermagnesemia caused by magnesium oxide, one of the laxatives, based on chronic renal failure.

## Case presentation

An 87-year-old woman was admitted to Tokuyama Medical Association Hospital because of two syncopal attacks within past 2 days, from which she recovered in a few minutes without residua. She had been treated for a long time for chronic renal failure, of which cause was suspected to be hypertensive and arteriosclerotic renal disease without diabetes mellitus. She had been given with calcium polystyrene sulfonate 4 g, azocemide 30 mg, benidipine hydrochloride 8 mg, telmisartan 40 mg, doxazosin mesylate 0.5 mg, and febuxostat 10 mg every day for hypertension and renal failure. Moreover, she had severe constipation, for which three kinds of laxatives had been given, including dioctyl sodium sulfosuccinate 90 mg mixed with casanthranol 45 mg, magnesium (Mg) oxide 660 mg (containing Mg 398 mg) and sennoside 12 mg every day, among which Mg oxide was started 4 months before.

On admission, her blood pressure was 113/84 mmHg with heart rate 78/min, regular. She looked somewhat obtunded, although she showed correct orientation and ordinary conversations with mild dysarthria and was judged to have normal consciousness. She could not stand up and walk due to mild muscle weakness of the lower limbs. Electrocardiography (ECG), chest x-ray studies, and Holter ECG for 24 h were all within normal limits. Head magnetic resonance imaging (MRI) computed tomography, including diffusion-weighted modality, revealed a few old lacunar infarctions in the white matter of the cerebral hemispheres, without abnormal intensity lesions in the basal ganglia and frontal lobes. Electroencephalography (EEG) disclosed diffuse and slow wave (6–7 Hz) basic activities, suggesting cerebral hypofunction rather than an impaired consciousness. Laboratory examination of serum on hospital day 1 disclosed a decrease in albumin (3.5 g/dL) and an increase in blood urea nitrogen (49.0 mg/dL), creatinine (2.59 mg/dL), K (5.2 mEq/L) with a decrease in eGFR (14.0 mL/min/1.73 m^2^) (Table 2), a slight increase in C-reactive protein (CRP, 1.8 mg/dL) and normal aspartate transaminase (AST), alanine transaminase (ALT), γ-glutamyl transpeptidase (γ-GTP), Na, Cl and glucose concentrations. Complete blood counts (CBC) revealed a decrease in red blood cells (RBC, 298 × 10^4^/mL), hemoglobin (Hb, 9.8 g/dL), and hematocrit (Ht, 29.9%); an increase in white blood cells (WBC, 10.5 × 10^3^/mL) with increased stab and polymorphonuclear WBC percentage (80.5% in total); and normal platelet count. Urinary examination revealed mild proteinuria (< 100 mg/dL) without other abnormalities.

**Table 2:** Laboratory data of serum of the patient

	Normal range	Hospital day	1	10	17	24
		(unit)				
Urea N	8–22	(mg/dL)	49.0	101.2	104.8	55.2
Creatinine	0.5–1.1	(mg/dL)	2.59	3.10	3.14	1.95
Uric acid	2.5–6.5	(mg/dL)	*	8.5	7.8	*
eGFR	> 60	(mL/min/1.73 m^2^)	14.0	11.5	11.4	19.2
Na	136–148	(mEq/L)	138	138	138	141
K	3.6–5.0	(mEq/L)	5.2	6.9	5.5	5.3
Cl	98–110	(mEq/L)	100	105	109	116
Ca	4.5–5.5	(mEq/L)	*	4.4	4.3	4.2
Inorganic P	1.6–2.7	(mEq/L)	*	3.7	3.5	2.2
Mg	1.6–2.1	(mEq/L)	*	3.6	3.1	1.9
Glucose	70–109***	(mg/dL)	140**	122***	115***	*
HbA1c	4.6–6.2	(%)	*	6.1	6.3	*
Ammonium	30–80	(μg/dL)	*	30	*	*

*Not examined, **casual value, ***fasting value

Her hospital days were uneventful until the 10th hospital day, when she began to show queer involuntary movements. While she sat to eat meals holding chopsticks with the right hand, her right arm suddenly dropped. Then, she again elevated the right arm, followed by sudden drops and difficulty to eat. Her left arm also dropped when elevated. While she was sitting on the bed, her neck sometimes dropped forwards and the trunk fell ahead. When she was standing with help, her legs lost power suddenly, falling without aids. Therefore, she was obliged to be bed-ridden. She said the phenomena were very troublesome, although she could not tell whether she had experienced such abnormal involuntary movements in the past. On neurologic examination, it was clear that these symptoms were asterixis, and when her arms were kept elevated by an examiner, typical flapping tremor was seen in both hands. Her intellectual function was evaluated with the revised Hasegawa dementia scale (HDS-R) [4], which was verbal cognitive test known to be as useful as the mini-mental state examination (MMSE). In the clinical practice, an HDS-R score of 25–30 suggests normal cognition, 20–24 mild cognitive impairment, 15–19 mild dementia, 10–14 moderate dementia, and –9 severe dementia. Her score was 10, moderately low. She showed mild dysarthria. Deep tendon reflexes were decreased in the upper limbs and absent in the lower limbs without extensor plantar reflexes. Muscle tonus was normal in all limbs. Muscle strength was normal at the beginning, however, followed by transient loss of power due to asterixis. Sensory functions were obscure because the exact answers were not obtained.

In this case asterixis involving neck, trunk and all limb muscles were obvious, and because of lack of focal neurologic signs, metabolic causes were suspected of. Serum ammonium was 30 μg/dL (normal 30–80) on hospital day 10; therefore, hepatic failure and portal systemic shunt were excluded. At this time renal failure increased in severity because there were a more increase in serum urea nitrogen (101.2 mg/dL), creatinine (3.10 mg/dL), uric acid (8.5 mg/dL), and K (6.9 mEq/L) with decreased eGFR (11.5 mL/min/1.73 m^2^). CBC revealed a more decrease in RBC (263 × 10^4^/mL), Hb (8.9 g/dL), and Ht (26.4%), and a mild increase in WBC (9.7 × 10^3^/mL). Other serum electrolytes were examined at this time, revealing a slight decrease in Ca (4.4 mEq/L, normal 4.5–5.5), and an increase in inorganic P (3.7 mEq/L, normal 1.6–2.7) and Mg (3.6 mEq/L, normal 1.6–2.1).

**Fig. 1:**
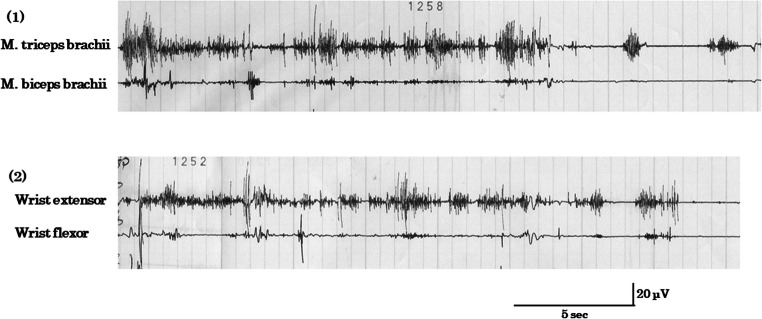
Surface EMG showing asterixis. (1) When the left arm was elevated voluntarily, there were irregular lapses of contractions of m. triceps brachii, resulting in arm dropping. M. biceps brachii showed no contractions meanwhile. (2) When the left forearm was kept elevated by an examiner, there appeared asterixis of the extended wrist and fingers, showing flapping tremor. The wrist flexor muscles showed no contractions

The cause of asterixis might be azotemia from the literature [1]. However, we considered hypermagnesemia would be more important. Mg oxide 660 mg per day had been given as one of the laxatives for the past 4 months, and it was withdrawn immediately after hypermagnesemia was detected. Thereafter, her asterixis gradually improved, and on the 17th hospital day asterixis of the neck and trunk disappeared, and upper and lower limbs dropped less frequently than before, and with mild flapping tremor of the fingers. At this time, serum Ca was 4.3 mEq/L, inorganic P 3.5 mEq/L, and Mg 3.1 mEq/L, still showing an increase in Mg concentration. Renal failure was unchanged in severity. Surface electromyography (EMG) exhibited asterixis of the arm muscles, because while the right arm was elevated, irregular lapses of the contractions of m. triceps brachii appeared, and while the forearm was kept elevated by an examiner, the loss of power of the wrist extensor muscles was demonstrated (Fig. 1). Based on these data, the most probable cause of asterixis seemed to be hypermagnesemia. Until the 24th hospital day, asterixis completely disappeared. However, she showed weakness of the hands with grip power 1 kg on the right and 3 kg on the left, and weakness of flexor and extensor muscles of the feet. There was hyperalgesia of bilateral hands and feet. Her gait needed assistance. At this time, serum Ca was 4.2 mEq/L, inorganic P 2.2 mEq/L, and Mg 1.9 mEq/L, showing a slight decrease in Ca concentration and normal inorganic P and Mg concentrations. Renal failure slightly improved because the serum urea nitrogen was 55.2 mg/dL, creatinine 1.95 mg/dL, K 5.3 mEq/L, and eGFR 19.2 mL/min/1.73 m^2^.

As to drug therapy after admission, calcium polystyrene sulfonate was increased from 4 g to 10 g every day on admission in order to lower serum K, and other drugs were not changed except for the withdrawal of magnesium oxide on the 10th hospital day. Concerning renal failure, hemodialysis therapy was explained to the patient and her family, although they did not ask it and rehabilitation alone was continued.

## Discussion

This case with known chronic renal failure developed suddenly systemic asterixis, involving the muscles of the neck, trunk, and all limbs. All of these muscles could not maintain their powers while keeping indicated postures. Neck suddenly dropped, trunk suddenly flexed ahead, and the limbs dropped when holding upward. Typical flapping tremor was seen bilaterally. Asterixis or negative myoclonus was demonstrated using surface EMG.

The exact mechanism of generation of asterixis remains unexplored [1, 2]. Most of the disorders to evoke asterixis are usually included in those suppressing the central nervous system [1, 2]. An impaired function of basal ganglia may play a role in the pathogenesis of asterixis. It was reported that there were functional or organic disturbances in the basal ganglionic region of the patients with hepatic encephalopathy using diffusion-weighted MR imaging and proton magnetic resonance spectroscopy (^1^H-MRS) [5]. In our case who underwent MR study including diffusion-weighted MR imaging, no abnormities were observed in the basal ganglia, although MRS investigations were not performed.

There is another hypothesis that abnormal function of diencephalic motor centers that regulate the agonist and antagonist tones may be essential [6]. However, its clear certification has not been hitherto obtained with electrophysiological studies or other methods. Clinically, liver failure or hyperammonemia was the most popular cause of asterixis, which was denied in our case because of normal concentration of serum ammonium. Azotemia may be the second possibility in our case (Table 1), in whom renal failure had persisted several years with marked azotemia. And, when asterixis disappeared 14 days later, renal function had somewhat improved. However, the factor most related to the occurrence of asterixis was considered to be elevated serum Mg from Mg oxide in laxatives, of which value was elevated (3.6 mEq/L; normal 1.6–2.1) at the beginning of asterixis and decreased to normal (1.9 mEq/L) when asterixis disappeared. Renal failure definitely caused the elevation of serum Mg, and might have contributed to the augmentation of asterixis itself.

The levels of elevated serum Mg concentration are known to be closely related to the severity of clinical features [3]: (1) mild elevation (< 5.8 mEq/L): asymptomatic, weakness, nausea, or confusion; (2) moderate elevation (5.8–9.8 mEq/L): worsening of confusional state, bladder paralysis, slight reduction in blood pressure, bradycardia, or blurred vision; (3) severe elevation (> 9.8 mEq/L): muscle flaccid paralysis, hypotension and bradycardia, atrioventricular block or lethargy. And at more than 12 mEq/L, cardiopulmonary arrest may occur [3]. In Japanese literatures, the toxic level of hypermagnesemia was described to be 4 mEq/L or more, and above 4 mEq/L hypotension, skin flush, nausea, vomiting, and/or bradycardia may occur [7]. In our case, the maximum of elevated serum Mg concentration was 3.6 mEq/L with only mild increase. Severe asterixis of the whole body gradually decreased in severity along with the decreasing Mg concentration, meaning Mg toxicity was probably the most important cause in our case. Many drugs suppressing the functions of the central nervous system and muscles may cause asterixis (Table 1), all of which were not used in our case.

While asterixis appeared, serum Ca concentration was slightly decreased (4.3–4.4 mEq/L) and inorganic P was slightly increased (3.5–3.7 mEq/L). Hypocalcemia may be the causes of neuromuscular hyperexcitability, such as tetany and seizure, and hyperphosphatemia itself is usually asymptomatic although it is a risk factor of cardiovascular diseases [8]. There have been no reports that hypocalcemia or hyperphosphatemia played a role in the pathogenesis of asterixis. As to the abnormal involuntary movements caused by hypermagnesemia, there was a description that when associated with hypocalcemia, hypermagnesemia may induce choreiform movements and seizures [3].

Additionally, this case showed absence of deep tendon reflexes in the lower limbs and distal-dominant muscle weakness and hyperalgesia after the cessation of asterixis. This was probably sign of polyneuropathy due to chronic renal failure, although the electrophysiological studies were not conducted. We reported a case of asterixis lasting 2 weeks probably due to hypermagnesemia from taking Mg oxide in chronic renal failure.
